# A Review of Experimental Studies on Natural Chalcone-Based Therapeutic Targeting of Genes and Signaling Pathways in Type 2 Diabetes Complications

**DOI:** 10.3390/genes15070942

**Published:** 2024-07-18

**Authors:** Naser A. Alsharairi

**Affiliations:** Heart, Mind and Body Research Group, Griffith University, Gold Coast, QLD 4222, Australia; naser.alsharairi@gmail.com

**Keywords:** diabetes mellitus, microvascular complications, macrovascular complications, chalcones, gene expression, signaling pathways

## Abstract

Diabetes mellitus type 2 (T2DM) is a common chronic condition that presents as unsettled hyperglycemia (HG) and results from insulin resistance (IR) and β-cell dysfunction. T2DM is marked by an increased risk of microvascular and macrovascular complications, all of which can be the cause of increasing mortality. Diabetic nephropathy (DNE), neuropathy (DNU), and retinopathy (DR) are the most common complications of diabetic microangiopathy, while diabetic cardiomyopathy (DCM) and peripheral vascular diseases are the major diabetic macroangiopathy complications. Chalcones (CHs) are in the flavonoid family and are commonly found in certain plant species as intermediate metabolites in the biosynthesis of flavonoids and their derivatives. Natural CHs with different substituents exert diverse therapeutic activities, including antidiabetic ones. However, the therapeutic mechanisms of natural CHs through influencing genes and/or signaling pathways in T2DM complications remain unknown. Therefore, this review summarizes the existing results from experimental models which highlight the mechanisms of natural CHs as therapeutic agents for T2DM complications.

## 1. Introduction

Diabetes mellitus type 2 (T2DM) is a chronic metabolic condition causing morbidity and mortality worldwide [1,2]. Around 445 million people were living with T2DM in 2020, and this number is estimated to have increased to 730 million by 2050 [3]. T2DM is mostly seen in adults [4], and it is marked by dysregulated pancreatic insulin secretion, insulin resistance (IR), hyperglycemia (HG), and hyperinsulinemia (HI), which all trigger reactive oxygen species (ROS) production, oxidative/metabolic stress, endoplasmic reticulum (ER) stress, inflammatory stress, and amyloid stress [5,6,7]. Several risk factors associated with T2DM include genetic predisposition, unhealthy diet, physical inactivity, cardiovascular disease (CVD), dyslipidemia (DLP), hypertension (HTN), high C-reactive protein (CRP), depression, watching TV, smoking, and air pollution [7,8,9]. T2DM complications can be categorized into microvascular and macrovascular complications. The most common T2DM microvascular complications are diabetic nephropathy (DNE), neuropathy (DNU), and retinopathy (DR). Diabetic cardiomyopathy (DCM), ischemic heart disease, and stroke are the major T2DM macrovascular complications [10,11,12,13]. Recently, omics technologies, including transcriptomics, proteomics, metabolomics, microbiomics, epigenomics, and genomics, have been utilized to identify multiple molecular mechanisms and signaling pathways behind T2DM complications [14,15,16,17].

Chalcones (CHs) are intermediate metabolites found in certain plant species (e.g., *Glycyrrhiza*, *Scutellaria*, *Humulus*) that have a significant role in the biosynthesis of flavonoids and their derivatives [18,19]. Isoliquiritigenin (ILTG), licochalcones (Licos), xanthohumol (XN), butein (BU), cardamonin (CAD), and panduratin (PA) are among the natural CHs with therapeutic potential against several diseases such as cancer, obesity, and diabetes [19,20,21]. CHs with chloro, iodo, bromo, hydroxy, methoxy, and benzyloxy substitutions at positions on the A and/or B rings exhibit a high-glucose-lowering effect in vitro [22]. An in vivo experiment demonstrated significant reductions in HG and fasting glucose levels in mice treated with CH derivatives substituted by hydroxyl and methoxy groups at positions 2′, 4′, and 5 on the benzaldehyde and acetophenone rings [23]. CH derivatives synthesized from acetophenon and aromatic aldehyde showed high antidiabetic potential in diabetic rats by reducing the blood levels of glucose, triglycerides, and creatinine [24]. The hydrophobic and hydroxyl groups attached to the CHs skeleton improve antidiabetic activities by modulating therapeutic targets such as dipeptidyl peptidase 4 (DPP-4), α-glucosidase, α-amylase, adenosine monophosphate-activated protein kinase (AMPK), and peroxisome proliferator-activated receptor-γ (PPARγ) [25,26,27].

Despite several experiments on CHs as potential targets for T2DM treatment [25,26,27,28], there is no review of experimental studies exploring the mechanisms of natural CHs for the treatment of T2DM complications. Therefore, this review highlights the existing experiments focusing on the therapeutic mechanisms of all natural CHs in T2DM complications.

## 2. Methods

**Figure 1:**
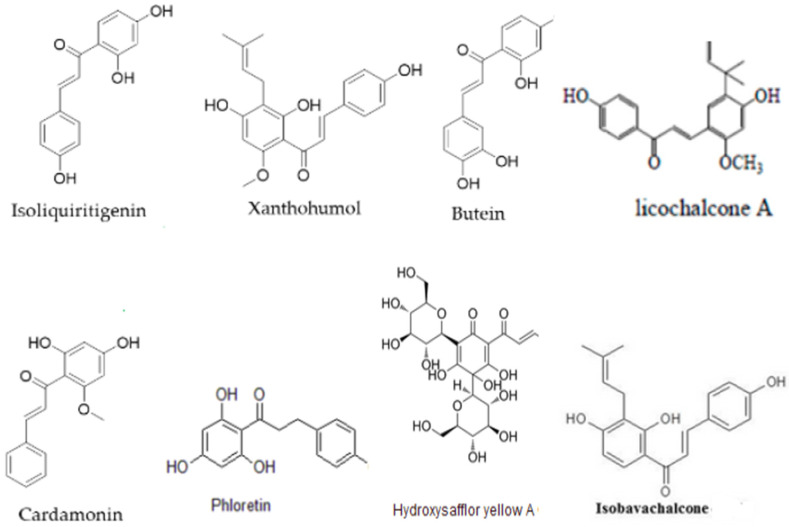
Chemical structures of natural CHs [18,21,28].

The PubMed/Medline database was searched up to 15 June 2024 to extract English-language publications exploring the mechanisms of natural CHs as therapeutic agents for T2DM complications. The Boolean operator “AND” was utilized to combine the following search terms: “ILTG”, “Licos”, “XN”, “BU”, “CAD”, “PA”, “isobavachalcone” (ISO) “hydroxysafflower yellow A” (HSYA), “phloretin” (PH), 4-hydroxyderricin (4-HD), 4-methoxychalcone (4-ME), garcinol (GA), “DNE”, “DNU”, “DR”, “DCM”, “cardiac myocytes” (CM), “myocardial infarction” (MI), and “atherosclerosis”. The search was limited to studies reporting experimental research. Experiments related to synthesized CH derivatives and not focused on T2DM complications or even the mechanisms of natural CHs in T2DM complications were excluded. A total of 114 articles were identified; of these, 32 experiments were selected. These experiments demonstrated that ILTG, XN, LicoA, PH, HSYA, ISO, CAD, and BU could be effective in the treatment of T2DM complications. The structure of these CHs is shown in Figure 1.

## 3. The Therapeutic Mechanisms of Natural CHs in T2DM Microvascular Complications

### 3.1. Diabetic Nephropathy

Several experiments have shown that natural CHs, including ILTG, XN, LicoA, PH, HSYA, ISO, CAD, and BU, alleviate DNE via altering the genes/signaling pathways of renal fibrosis, proliferation, angiogenesis, apoptosis, inflammation, and oxidative stress. The treatment of human renal mesangial cells under conditions of high glucose with nontoxic ILTG (≥10 μM) inhibited glomerulosclerosis/mesangial fibrosis and mesangial cell proliferation by reducing type IV collagen production and the expression of tissue inhibitor of matrix metalloproteinases-2 (TIMP-2) and cellular connective tissue growth factor (CTGF) through repressing the transforming growth factor β (TGF-β)-mothers against decapentaplegic homolog (SMAD) signaling pathway [29]. ILTG treatment at 100 and 200 nM concentrations showed a significant inhibition of advanced glycation end product (AGE)-induced tubular epithelial–myofibroblast transdifferentiation and collagen secretion in renal proximal tubule cells through downregulating vimentin levels, signal transducer and activator of transcription 3 (STAT3) phosphorylation, and the TGF-β/SMAD/STAT3 signaling pathway [30]. Oral administration of ILTG reduces renal damage in diabetic rats by downregulating oxidative stress markers and renal inflammatory cytokines [31]. ILTG was reported to inhibit high-glucose-induced proliferation and inflammation and the accumulation of the extracellular matrix (ECM) in mouse glomerular mesangial cells when treated at a concentration of 20 μM. This could be related to a decrease in the mRNA levels of pro-fibrotic markers and proinflammatory cytokines through suppressing the Janus kinase 2 (JAK2)/STAT3 signaling pathway [32]. ILTG effectively inhibited the viability of high-glucose-induced renal cells when treated at a dose of 20–80 μg/mL through ameliorating oxidative stress markers. ILTG also improved renal morphology and oxidation stress markers while suppressing fibrosis-related factors and the JAK2/STAT3 signaling pathway in streptozotocin (STZ)-induced diabetic rats [33].

An experiment showed that XN reduces blood glucose levels and modulates the vascular endothelial growth factor (VEGF) angiogenic pathway in the kidneys of diabetic mice by reducing phospho-vascular endothelial growth factor receptor-1/2 (p-VEGFR-1/2) expression and its downstream effectors [34]. It has been shown that oxidative stress reduces in the kidneys of diabetic mice supplemented with XN by decreasing the accumulation of AGE and galectin-3 (Gal3) expression [35]. XN treatment at a dose of 50 μM reduced high-glucose-induced oxidative stress in human kidney tissues and STZ-induced kidney injury in diabetic mice. This is mediated by decreasing ROS levels and increasing catalase (CAT)/SOD activity, as well as by the mRNA levels of nuclear factor erythroid 2-related factor 2 (Nrf2) and its downstream genes [36].

LicoA treatment reduces DNE in diabetic mice through downregulating and/or upregulating of proteins expression and oxidative stress parameters [37]. Treatment with PH at a low concentration (20 mg kg^−1^) significantly alleviated podocyte injury in diabetic rats through restoring podocin and nephrin expression [38]. An experiment carried out on STZ and high-fat-diet-induced DNE in mice showed that HSYA treatment at a concentration of 120 mg/kg attenuates renal cell apoptosis, oxidative stress, and inflammation, as evidenced by increased and/or decreased apoptotic protein expression and by inflammatory and oxidative stress markers in the kidneys [39]. HSYA treatment at a dose of 100–200 μM was shown to reduce HG-induced inflammation and podocyte apoptosis in mouse macrophage cells by decreasing the protein and mRNA levels of inflammatory and oxidative stress genes while increasing mannose receptor 206 (CD206) and arginase-1 (Arg-1) protein expression [40]. In vitro and in vivo experiments showed that HYSA treatment at a concentration of 10 mg/kg decreases renal fibrosis in human renal glomerular endothelial cells and STZ-induced diabetic rats by ameliorating oxidative stress/inflammatory markers and downregulating the Toll-like receptor 4/nuclear factor-κB (p65) (TLR4/NF-κB p65) signaling pathway [41].

ISO treatment attenuates high-glucose-induced endothelial damage in vitro renal glomerular endothelial cells model, as well as suppresses renal inflammation and glomerular endothelial cell apoptosis in STZ-induced DNE rats by reducing the expression of inflammatory cytokines through blocking the activation of NF-κB signaling pathway [42]. It has been reported that CAD treatment at different concentrations reduces kidney damage in methylglyoxal (MGO)-treated rat kidney tubular epithelial cells through altering DNE-mediated gene expression and signaling pathways [43]. Treatment with BU showed anti-diabetic activity and normal morphology of glomerulus and renal tubular in STZ-induced diabetic rats without any side effects through the downregulation of PPARγ expression [44].

### 3.2. Diabetic Neuropathy

A few in vivo experiments have shown that ILTG and PH modulate gene expression of autophagy, inflammation, and oxidative stress in a rat model of DNU. ILTG administration to diabetic rats and neuro2a cells promoted Sirtuin (SIRT1) activity and NAD+/NADH ratio in peripheral sciatic nerves, resulting in alleviated neuropathic pain, decreased ROS production, and increased Nrf2 activity through upregulating Forkhead box O3 (FOXO3) signaling, peroxisome proliferator-activated receptor-γ coactivator 1 α (PGC-1α)-mediated mitochondrial biogenesis, and AMPK-mediated autophagy, along with the suppressing of mammalian target of rapamycin (mTOR) phosphorylation [45]. The combinational treatment of sciatic nerve tissue with PH and duloxetine (an antidepressant drug) was shown to decrease inflammatory and oxidative stress markers in diabetic rats [46].

### 3.3. Diabetic Retinopathy

The few experiments that have been performed suggest that XN, HSYA, PH, and BU attenuate DR by altering the gene expression and/or signaling pathways of oxidative stress inflammation, autophagy, apoptosis, and angiogenesis. XN treatment was shown to inhibit angiogenesis and regulate autophagy dysregulation in hypoxia and high-glucose-induced human retinal microvascular endothelial cells by downregulating and/or upregulating the protein and mRNA levels of angiogenesis and oxidative stress markers through the inhibition of PI3K/AKT/mTOR signaling pathway [47]. HSYA treatment was found to improve DR and attenuate retinal ganglion cell apoptosis in diabetic rats due to a mechanism involving increased and/or decreased inflammatory and oxidative stress markers [48]. PH treatment at a concentration of 20 mg/kg significantly improved DR in diabetic mice by upregulating and/or downregulating the expression of proteins involved in energy metabolism, apoptosis, and oxidative stress [49]. PH treatment improves human retinal pigment epithelial cells through reducing glucose uptake, inhibiting JNK phosphorylation and lipopolysaccharide (LPS)-induced proinflammatory cytokine production, and upregulating Nrf2 activity [50]. The findings of an in vitro experiment showed that BU treatment at different concentrations inhibits fluorescent AGEs and α-crystallin-induced lens proteins aggregation [51].

Table 1 presents the therapeutic mechanisms of natural CHs in T2DM microvascular complications.

## 4. The Therapeutic Mechanisms of Natural CHs in T2DM Macrovascular Complications

Results from a few experiments demonstrated that ILTG, HSYA, and PH reduce DCM, diabetic atherosclerosis (DAS), and diabetic vascular injury (DVI) through the modulation of multiple genes.

### 4.1. Diabetic Cardiomyopathy

An experiment using embryonic rat heart cells demonstrated the inhibition of high-glucose-induced apoptosis, fibrosis, and hypertrophy following treatment with ILTG at different concentrations via ameliorating inflammatory and oxidative stress markers at the mRNA and protein levels. ILTG treatment was also found to attenuate apoptosis/heart fibrosis and myocardial inflammation and oxidative stress in diabetic rats [52]. HSYA administration to diabetic mice supplemented with STZ and a fat diet demonstrated antioxidant effects against DCM by regulating the balance between ROS and antioxidant enzymes [53]. Oral administration of PH to *db/db* mice protected against DCM by altering proteins involved in cardiac lipid metabolism and myocardial mitochondria [54]. PH was demonstrated to exhibit inhibitory effects on HG-induced fibrosis injury, cardiomyocyte oxidation, and hypertrophy in cardiac cells when treated at different concentrations, with no toxic effect observed. PH treatment also effectively prevented cardiomyocyte injury and suppressed cardiac oxidative stress/fibrosis in diabetic mice via the downregulation of Kelch-like ECH-associated protein 1/Nrf2 (Keap1/Nrf2) signaling pathways [55]. Treatment with PH at different concentrations showed effective in vitro results in attenuating HG-induced inflammation, apoptotic death, and fibrotic response in cardiomyocytes and preventing cardiomyocyte injury, as well as inhibiting cardiomyocyte inflammatory response in STZ-induced diabetic mice. These effects were mediated by the amelioration of inflammation, fibrosis, and hypertrophy-related genes at the mRNA and protein levels via upregulating SIRT1 expression [56].

### 4.2. Diabetic Atherosclerosis

HSYA has exerted antioxidant and anti-ferroptosis effects in diabetic mice and human umbilical vein endothelial cells by reducing atherosclerotic plaque formation via the regulation of ferroptosis and oxidative stress-related markers [57]. PH exerts a therapeutic effect against HG-induced endothelial cell dysfunction and decreases the acceleration of atherosclerosis in diabetic mice treated at a dose of 20 mg/kg through the upregulation of nitric oxide synthase (eNOS) and kruppel-like factor 2 (KLF2) expression [58].

### 4.3. Diabetic Vascular Injury

An in vitro experiment showed that HSYA at a concentration of 50 μM reduces high-glucose-induced vascular injury by decreasing endothelial cell apoptosis, monocyte adhesion, vascular permeability, oxidative stress markers, and cell adhesion molecule (CAM) levels [59]. PH has demonstrated the effects of alleviating high-glucose/AGE-induced vascular injury and the EndMT process in human umbilical vein endothelial cells following treatment at a nontoxic concentration of 40 μM through regulating the protein and mRNA levels of mesenchymal/endothelial markers. Furthermore, PH has the potential to reduce vascular fibrosis and endothelial damage in diabetic mice by downregulating the mRNA levels of TGFβ, intercellular adhesion molecule (ICAM), bone morphogenetic protein-2 (BMP2), and monocyte chemoattractant protein-1 (MCP1) in aorta tissues [60].

The mechanisms of natural CH treatment for T2DM microvascular complications is presented in Table 2.

## 5. Conclusions

T2DM is a disease characterized by HG and resulting from increased pancreatic β-cell dysfunction and IR. The literature has demonstrated that T2DM causes several micro-and macrovascular complications via multiple gene expression and signaling pathways. However, the specific mechanisms of natural CHs through modulating gene expression/signaling pathways and the protective role thereof in T2DM complications remain unclear and mostly unexplained.

Several experiments, which mostly targeted rat/mouse models in vivo and human renal mesangial cells in vitro, showed that ILTG, XN, LicoA, PH, HSYA, ISO, CAD, and BU alleviate DNE through modulating gene expression/signaling pathways of inflammation, oxidative stress, proliferation, apoptosis, angiogenesis, and renal fibrosis [29,30,31,32,33,34,35,36,37,38,39,40,41,42,43,44]. A few experiments showed that ILTG and PH relieve oxidative stress, inflammation, and autophagy in rat models, possibly mediated via modulating a number of genes/signaling pathways responsible for DNU development [45,46]. Other findings from a few experiments in diabetic mice and human retinal microvascular endothelial cells revealed that XN, HSYA, PH, and BU reduce inflammation, oxidative stress, angiogenesis, autophagy, and apoptosis through regulating DR-related genes and/or signaling pathways [47,48,49,50,51].

Natural CHs, and ILTG, HSYA, and PH, in particular, act on multiple targets of macrovascular T2DM complications and regulate signaling pathways/genes, resulting in protection against DCM, DAS, and DVI. A few experiments in rat embryonic heart cells and diabetic rats have confirmed the potential of ILTG, HSYA, and PH in ameliorating HG-induced DCM through reducing oxidative stress, inflammatory/fibrotic responses, and apoptotic death [52,53,54,55,56]. The limited evidence available in diabetic rats and human umbilical vein endothelial cells suggests the efficacy of HSYA and PH in ameliorating DAS and DVI-associated oxidative stress and ferroptosis [57,58,59,60].

## 6. Future Directions

Further experiments are warranted to explore the role of natural CHs in the treatment of T2DM complications through the regulation of multiple genes and/or signaling pathways involved in inflammation, oxidative stress, angiogenesis, apoptosis, and autophagy. Both in vivo and in vitro experiments demonstrated antidiabetic effects, but the ideal concentration in treatment still needs more investigation.

A few experiments indicate that ILTG and PH protect against DNE, DCM, and DVI, with no toxic effect observed. More experiments are needed to determine if natural CHs have any toxic effects on human/rat cells at concentrations that have shown efficacy in treating T2DM complications. The effects of natural CH-rich extracts from plants on T2DM complications need to be further analyzed in human and animal models. Proteomics, transcriptomics, and metabolomics are among the omics technologies targeting T2DM complications and are used as single techniques or are combined in experiments. There is a need for further experiments utilizing multi-omics technologies to determine the underlying cellular pathways and molecular genes of T2DM complications at different dimensions. Only a few human experiments suggest that natural CHs alleviate T2DM complications. Further human experimental and clinical trials are needed to explore the role of natural CHs as therapeutic agents in T2DM complications.

## Figures and Tables

**Table 1:** Summary of experimental models focusing on natural CHs for the treatment of microvascular T2DM complications.

Microvascular Complications	Model Type	Omics Technique(s)	Natural CHs	Treatment	Outcomes	Mechanisms of Action	Ref.
DNE	Human renal mesangial cells	Transcriptomics	ILTG	Treatment of cells with ILTG at 1–20 μM concentrations and incubation for 3 days	Glomerulosclerosis, mesangial fibrosis, mesangial cell proliferation ↓	Genes/proteins: CTGF, TIMP-2, MT-1 MMP, TGF-β, SMAD-4/7, type IV collagen ↓ Pathways: SMAD- TGF-β ↓	[29]
DNE	Human proximal tubular cells	Proteomics	ILTG	Treatment of cells with ILTG at 50–200 nM concentrations and incubation for 48 h	Renal tubular fibrosis ↓	Genes/proteins: p-STAT3, vimentin ↓ Pathways: TGF-β/STATS3/SMAD ↓	[30]
DNE	Male Sprague Dawley diabetic rats	Proteomics, metabolomics	ILTG	Rats were randomized into normal group (received carboxymethyl cellulose) and diabetic group (received ILTG at dose equals 20 mg/kg/day) for 8 weeks	Renal oxidative stress and inflammation ↓	Genes/proteins: IL-10 ↑; Sirt-1, TNF-α, IL-1β, NLRP3 ↓ Antioxidant enzymes/lipid peroxidation: GSH, TAC ↑; MDA ↓ Pathways: Sirt-1/NFκB ↓	[31]
DNE	Mouse glomerular mesangial cells	Transcriptomics	ILTG	Treatment of cells with ILTG at 20 μM concentration and incubation for 48 h	Glomerular mesangial cell inflammation, proliferation, and ECM ↓	Genes/proteins: fibronectin, collagen IV, the mRNA levels of TNF-α, IL-1β, rIL-6, TGF-β1, CTGF ↓ Pathways: JAK2/STAT3 ↓	[32]
DNE	Human renal cells, female Sprague Dawley diabetic rats	Proteomics	ILTG	Treatment of cells with ILTG at 20, 40, and 80 μg/mL concentrations and incubation for 24 h Rats were randomized into 4 groups, namely a saline-treated control and three groups that received 10, 20, and 40 mg/kg ILTG	Renal fibrosis, inflammation, and oxidative stress ↓	Genes/proteins: collagen-1, fibronectin, TGF-β1 ↓ Oxidants/antioxidant enzymes: SOD2, GPX1, GSH ↑; ROS ↓ Pathways: JAK2/STAT3 ↓	[33]
DNE	Male C57BL/6 diabetic mice	Proteomics	XN	Mice were fed normal diet (group 1), fat diet (group 2), fat diet and 0.1% ethanol in drink water (group 3), fat diet and 10 mg/L XN (group 4), and fat diet and 10 mg/L 8-Prenylnaringenin (group 5) for 20 weeks	Angiogenesis in kidney ↓	Genes/proteins: p-VEGFR-1/2, Akt, ERK, NP-1, PFKFB3 ↓ Pathways: VEGF ↓	[34]
DNE	Male C57BL/6 diabetic mice	Proteomics, metabolomics	XN	Mice were fed normal diet (control group) and fat diet with XN or 8-Prenylnaringenin (diabetic group) for 20 weeks	Renal oxidative stress ↓	Genes/proteins: Gal3 ↓ Metabolites: AGE ↓	[35]
DNE	Human renal glomerular endothelial and kidney cells, male C57BL/6 diabetic mice	Transcriptomics	XN	Treatment of cells with XN at 50 μM concentrations and incubation for 48 h Mice were assigned to control group (saline plus DMSO), XN group (25 mg/kg XN in saline plus DMSO), DNE group (STZ plus DMSO), and DNE plus XN group (STZ plus 25 mg/kg XN in saline plus 0.5% DMSO)	Renal oxidative stress ↓	Oxidants/antioxidant enzymes: CAT, SOD, Nrf2 ↑; ROS, HMOX1, NQO1 ↓	[36]
DNE	Male C57BL/6 diabetic mice	Proteomics	LicoA	Mice were fed normal diet (control group) and fat diet (diabetic group) for 12 weeks	Oxidative stress and kidney damage ↓	Genes/proteins: GLUT1, TGF-β1, HIF-1α, RAGE, AGEs ↓ Oxidants/lipid peroxidation: GSH-Px, SOD, Nrf2 ↑; MDA ↓	[37]
DNE	Male C57BL/6 *ApoE*^−/−^ diabetic mice	Proteomics	PH	Mice were treated with STZ (50 mg kg^−1^) for 5 days and a cholesterol-rich diet for 6 weeks, and then with PH (20 mg kg^−1^) daily for one week	Renal damage ↓	Genes/proteins: Podocin, nephrin ↑	[38]
DNE	Wistar rats	Proteomics, metabolomics	HSYA	Rats were orally administered HSYA at a dose of 120 mg/kg for 8 weeks	Renal oxidative stress, inflammation, and apoptosis ↓	Genes/proteins: Bcl-2 ↑; Bax, caspase-3, TNF-α, LDH ↓ Antioxidant enzymes/lipid peroxidation: SOD ↑; MDA ↓ Metabolites: FFA ↓	[39]
DNE	Mouse RAW264.7 macrophage cell line	Proteomics, transcriptomics	HSYA	Treatment of cells with HSYA at 100 and 200 μM concentrations and incubation for 24 h and 48 h	Podocyte apoptosis ↓	Genes/proteins: the protein and/or mRNA levels of Arg-1, CD206 ↑; TNF-α, iNOS ↓	[40]
DNE	Human immortalized renal tubular epithelial cells, male Sprague Dawley diabetic rats	Proteomics, transcriptomics	HSYA	Treatment of cells with HSYA (10 mg/mL) Rats were administered 55 mg/kg STZ and then divided into diabetic and HSYA (10 mg/kg/d) groups	Renal fibrosis ↓	Genes/proteins: the protein and/or mRNA levels of T-AOC ↑; IL-6, TNF-α, Notch2, TLR4, NLRP3, NF-κB(p65), collagen IV ↓ Lipid peroxidation: MDA ↓	[41]
DNE	Human renal glomerular endothelial cells, male Sprague Dawley rats	Proteomics	ISO	Treatment of cells with ISO at 10 μM concentrations and incubation for 48 h Rats were supplemented with 60 mg/kg STZ and then divided into three groups, namely control group (25 mg/kg/d), DNE group (12.5 mg/kg/d), and ISO group (50 mg/kg/d)	Renal damage, inflammation, and apoptosis ↓	Genes/proteins: TNF-α, IL-1β, IL-6, p-IKKb, p-IκBα, p-p65, NF-κB ↓	[42]
DNE	Rat kidney tubular epithelial cells	Proteomics, metabolomics	CAD	Cells were divided into 5 groups, namely control group without treatment, MGO plus 10 μM CAD, MGO plus 20 μM CAD, MGO plus 40 μM CAD, and MGO plus 10 μM HC-030031	Renal damage, inflammation, and apoptosis ↓	Genes/proteins: Bcl-2, E-cadherin ↑; Bax, c-caspase-3, IL-6, IL-1β, TNF-α, Vimentin, TGF-β1, p65 ↓ Antioxidant enzymes/lipid peroxidation: GSH, SOD ↑; MDA ↓ Metabilites: AGEs, RAGE ↓ Pathways: PI3K/AKT, JAK/STAT ↓	[43]
DNE	Wistar diabetic rats	Proteomics	BU	Rats were assigned to 5 groups, namely control group (0.1 M citrate buffer pH 4.5), diabetic group (STZ 65 mg/kg b.w plus 110 mg/kg of nicotinamide), BU group (2.5 mg/Kg b.w), chitosan microparticle group (2.5 mg/Kg b.w), and metformin group (5 mg/Kg b.w)	Kidney functions ↑	Genes/proteins: PPARγ ↓	[44]
DNU	Neuro2a neuroblastoma cells, male Sprague Dawley diabetic rats	Proteomics	ILTG	Treatment of cells with ILTG at 30 nM concentrations and incubation for 24 h Rats were treated with ILQ at 10 and 20 mg/kg (STZ-D+ILQ-10/20)	Neuropathic pain ↓, mitochondrial biogenesis and autophagy ↓	Genes/proteins: SIRT1, NAD+/NADH, FOXO3, PGC-1α, AMPK ↑; mTOR ↓ Oxidants: Nrf2, ROS ↓	[45]
DNU	Male Wistar rats	Proteomics	PH	Rats were randomized into non-diabetic control group, STZ-induced DNU group, DNU group treated with 30 mg/kg/d duloxetine, DNU group treated with 15 mg/kg/d duloxetine, DNU group treated with 50 mg/kg/d PH, and DNU group treated with 25 mg/kg/d PH	Sciatic nerve inflammation and oxidative stress ↓	Genes/proteins: TNF-α, IL-6 ↓ Oxidants/antioxidant enzymes/lipid peroxidation: GSH ↑; MDA, NO ↓	[46]
DR	Human retinal microvascular endothelial cells	Transcriptomics	XN	Treatment of cells with XN at 0–80 μM concentrations and incubation for 24 h and 48 h	Retinal angiogenesis, autophagy, and oxidative stress ↓	Genes/proteins: PEDF, p62 ↑; the mRNA levels of HIF-1α, VEGF, LC3-II/I, Beclin-1, ATG5 ↓ Oxidants/antioxidant enzymes/lipid peroxidation: SOD ↑; ROS, MDA ↓ Pathways: PI3K/AKT/mTOR ↓	[47]
DR	Wistar diabetic rats	Proteomics	HSYA	Rats were assigned to control group (without treatment), model group treated with 60 mg/kg/d STZ, and HSYA group treated with 50 mg/kg/d HSYA daily for 6 weeks	Retinal ganglion cell apoptosis, inflammation, and oxidative stress ↓	Genes/proteins: Bcl-2 ↑; TNF-α, IL-1β p53 ↓ Antioxidant enzymes/lipid peroxidation: Nrf-2, HO-1, SOD ↑; MDA ↓	[48]
DR	C57BLKS/J *db/db* diabetic mice	Proteomics	PH	Rats were orally administered PH at a dose of 20 mg/kg for 10 weeks	Retinal cell apoptosis and oxidative stress ↓	Genes/proteins: Glr×-3 ↑;AGE, FBG, GFAP, γ-crystallin ↓	[49]
DR	Human retinal pigment epithelial cells	Proteomics	PH	Treatment of cells with PH at 100 μM and incubation for 72 h	Retinal inflammation ↓	Genes/proteins: IL-6, IL-8, VEGF, JNK ↓	[50]
DR	In vitro glycation of α-crystallin	Proteomics	BU	BU was used as a treatment at 25, 50, 100, and 200 μM concentrations and incubated for 7 days	Lens proteins aggregation ↓	Genes/proteins: AGEs, α-crystallin ↓	[51]

(↓) Decrease, (↑) increase.

**Table 2:** Summary of experimental models focusing on natural CHs for the treatment of macrovascular T2DM complications.

Macrovascular Complications	Models Type	Omics Technique(s)	Natural CHs	Treatment	Outcomes	Mechanisms of Action	Ref.
DCM	Embryonic rat heart-derived H9c2 cells, male C57BL/6 diabetic mice	Proteomics, transcriptomics	ILTG	Treatment of cells with ILTG at 2.5, 5, 10, 20, and 40 μM concentrations and incubation for 24 h Mice were supplemented with STZ at a concentration of 50 mg/kg/day for 5 days and then orally administered ILTG at a dose of 10 mg/kg and 20 mg/kg for 12 weeks	Cardiac apoptosis, hypertrophy, and fibrosis ↓ Myocardial oxidative stress and inflammation ↓	Genes/proteins: the mRNA and/or protein levels of Bcl-2 ↑; MAPKs, β-MyHC, ANP, TGF-β, collagen-1, Bax, TNF-α, IL-1β, MCP-1, ERK ↓ Oxidants: Nrf2, HO-1, NQO-1 ↑; ROS, H_2_O_2_ ↓	[52]
DCM	C57BL/6 diabetic mice	Metabolomics	HSYA	Mice were randomized into STZ and fat diet groups and then orally administered 60 mg/kg HSYA	Cardiac dysfunction and oxidative stress ↓	Oxidants/antioxidant enzymes/lipid peroxidation: GSH-Px, SOD ↑; MDA, ROS ↓	[53]
DCM	Male C57BLKS/J *db/db* and *db/m* diabetic mice	Proteomics	PH	Mice were assigned to diabetic group (without treatment) and PH group treated with 20 mg/kg PH for 10 weeks	Reduced DCM by altering genes involved in cardiomyopathy, mitochondrial function, and cardiac lipid metabolism	Genes/proteins: Mttp, Nampt, Ptpn11, LDLr, Ptplb, Sorbs1, Prkaa2, EndoG, Ndufs6, Ttn, DAPK3, Lama2, Lmna, Gsn, My12, Dmd, Ilk, Des, Glrx3, Col1a2 ↑; Gpihbp1, Atpaf2, Aifm2, Lias, Apc, Canx, Myom1, Cacnb2 ↓	[54]
DCM	H9c2 embryonic rat heart-derived cells, male C57BL/6 mice	Transcriptomics	PH	Treatment of cells with PH at 2.5, 5, 10, 20, 40, and 80 μM concentrations and 24 h incubation Mice were assigned to STZ group (100 mg/kg) and PH group (10 mg/kg)	Cardiomyocyte oxidation, cardiac hypertrophy and fibrosis ↓	Genes/proteins: LDH, AST, CK-MB, TGF-β, ANP, BNP, β-MyHC, CTGF, collagen-1, Keap1 ↓ Oxidants/antioxidant enzymes/lipid peroxidation: SOD, Nrf2, NQO-1, HO-1 ↑; ROS, MDA ↓	[55]
DCM	Rat myocardium myoblast (H9C2) cells, male C57BL/6 mice	Proteomics, transcriptomics	PH	Treatment of cells with PH at 2.5, 5, 10, 20, 40, and 80 μM concentrations and incubation for 24 h Mice were assigned to STZ group (50 mg/kg) and PH group (20 mg/kg)	Cardiac damage and inflammation, cardiomyocyte injury, apoptotic death, fibrotic injury ↓	Genes/proteins: the mRNA/protein levels of SIRT1 ↑; ANP, CK-MB, TGF-β, collagen-1, IκBα, IL-6, TNF-α ↓	[56]
DAS	Human umbilical vein endothelial cells, male *ApoE^−/−^* deficient C57BL/6 mice	Proteomics, transcriptomics	HSYA	Treatment of cells with HSYA at 25 μM concentration and incubation for 48 h Mice were supplemented with fat diet plus 30 mg/kg STZ and then injected with 2.25 mg/kg HSYA for 12 weeks	Aortic atherosclerotic plaques formation ↓ Ferroptosis and oxidative stress damage ↓	Genes/proteins: the protein and/or mRNA levels of SLC7A11 ↑; ACSL4, miR-429, ICAM, VCAM ↓ Oxidants/antioxidant enzymes/lipid peroxidation: GPX4, GSH-Px ↑; MDA, ROS ↓	[57]
DAS	Human umbilical vein endothelial cells, male *ApoE^−/−^* C57BL/6 mice	Proteomics, transcriptomics	PH	Treatment of cells with PH at 50 or 100 nM concentration and incubation for 48 h Mice were fed cholesterol-rich diet plus 50 mg/kg STZ and then injected with 20 mg/kg PH for 10 weeks	Atherosclerosis progression ↓	Genes/proteins: KLF2, eNOS ↑	[58]
DVI	Human umbilical vein endothelial cells	Proteomics	HSYA	Treatment of cells with HSYA at 0–50 μM concentrations and incubation for 24 h	Vascular permeability, monocyte adhesion, apoptosis, oxidative ↓	Genes/proteins: CAM, ICAM, VCAM, VEGF ↓ Oxidants: ROS, NOX4, H_2_O_2_ ↓	[59]
DVI	Human umbilical vein endothelial cells, male C57BL/6 mice	Transcriptomics	PH	Treatment of cells with PH at 10, 20, and 40 μM concentrations and incubation for 24 h Mice were supplemented with 100 mg/kg STZ for two days and then given 25 mg/kg/d and 75 mg/kg/d PH	Vascular fibrosis and endothelial damage ↓	Genes/proteins: ICAM-1, AMPK-PGC1α ↑; MCP1, BMP2, RANKL, vimentin, TGFβ ↓	[60]

(↓) Decrease, (↑) increase.

## Data Availability

Not applicable.

## Abbreviations

4-HD	4-hydroxyderricin
4-ME	4-methoxychalcone
ACSL4	Acyl-CoA synthetase long-chain family member 4
AGEs	Advanced glycation end products
Aifm2	ATP synthase mitochondrial F1 complex assembly factor 2
Akt	Threonine kinase
AMPK	Adenosine monophosphate activated protein kinase
ANP	Atrial natriuretic peptide
Apc	Adenomatous polyposis coli
Arg-1	Arginase-1
AST	Aspartate aminotransferase
ATG5	Autophagy related 5
Atpaf2	Apoptosis-inducing factor 2
Bax	Bcl-2-associated X protein
Bcl-2	B-cell lymphoma-2
BMP2	Bone morphogenetic protein-2
BNP	Brain natriuretic peptide
BU	Butein
Cacnb2	Voltage-dependent L-type calcium channel subunit β-2
CAD	Cardamonin
CAM	Cell adhesion molecule
Canx	Calnexin
CAT	Catalase
CD206	Mannose receptor 206
CHs	Chalcones
CI	Myocardial infarction
CK-MB	Creatine Kinase
CM	Cardiac myocytes
Col1a2	Collagen α-2(I) chain
CRP	High C-reactive protein
CTGF	Connective tissue growth factor
CVD	Cardiovascular disease
DAPK3	Death-associated protein kinase 3
DAS	Diabetic atherosclerosis
DCM	Diabetic cardiomyopathy
Des	Desmin
DLP	Dyslipidemia
Dmd	Dystrophin
DNE	Diabetic nephropathy
DNU	Diabetic neuropathy
DPP-4	Dipeptidyl peptidase 4
DR	Diabetic retinopathy
DVI	Diabetic vascular injury
ECM	Extracellular matrix
EndoG	Endonuclease G
ER	Endoplasmic reticulum
ERK	Extracellular-signal regulated kinase
FBG	Fasting blood glucose
Fe	Iron
FFA	Free fatty acid
FOXO3	Forkhead box O3
GA	Garcinol
Gal3	Galectin-3
GFAP	Glial fibrillary acidic protein
Glr×-3	Glutaredoxin-3
GLUT1	Glucose transporter type 1
Gpihbp1	Glycosylphosphatidylinositol-anchored high-density lipoprotein-binding protein 1
GPx	Glutathione peroxidase
GSH	Glutathione
Gsn	Gelsolin
H_2_O_2_	Hydrogen peroxide
HG	Hyperglycemia
HI	Hyperinsulinemia
HIF-α	Hypoxia-inducible factor-α
HMOX1	Heme oxygenase 1,HO-1
HO-1	Heme oxygenase-1
HSYA	Hydroxysafflower yellow A
HTN	Hypertension
ICAM	Intercellular adhesion molecule
IKKb	IkappaB kinase
IL	Interleukin
Ilk	Integrin-linked protein kinase
ILTG	Isoliquiritigenin
iNOS	Nitric oxide synthase
IR	Insulin resistance
ISO	Isobavachalcone
IκBα	Inhibitor of nuclear factor kappa B
JAK2	Janus kinase 2
Keap1	Kelch-like ECH-associated protein 1
KLF2	Kruppel-like factor 2
Lama2	Laminin subunit α-2
LC3	Microtubule-associated protein 1 light chain
LDH	Lactate dehydrogenase
LDLr	Low-density lipoprotein receptor
Lias	Lipoic acid synthase
Licos	Licochalcones
Lmna	Lamin A/C
LPS	Lipopolysaccharide
MAPKs	Mitogen activated protein kinases
MCP-1	Monocyte chemoattractant protein-1
MDA	Malondialdehyde
MGO	Methylglyoxal
miRNA	MicroRNA
MT-1	MMP Membrane type-1 matrix metalloproteinase
mTOR	Mammalian target of rapamycin
Mttp	Microsomal triglyceride transfer protein
My12	Myosin regulatory light chain 2
Myom1	Myomesin-1
Nampt	Nicotinamide phosphoribosyltransferase
Ndufs6	NADH dehydrogenase ubiquinone iron-sulfur protein 6
NFκB	Nuclear factor-κB
NLRP3	NOD-, LRR-, and pyrin domain-containing protein 3
NO	Nitric oxide
NOX4	NADPH oxidase 4
NP-1	Neuropilin-1
NQO-1	NADPH quinine oxidoreductase 1
Nrf2	Nuclear factor erythroid 2-related factor 2
p53	Protein 53
p62	Protein 62
p65	Protein 65
PA	Panduratin
PECAM-1	Platelet endothelial cell adhesion molecule-1
PEDF	Pre-autophagosomal structure
PFKFB3	Phosphofructokinase-2/fructose-2,6-biphosphatase 3
PGC-1α	Peroxisome proliferator-activated receptor-γ coactivator 1 α
PH	Phloretin
PPARγ	Peroxisome proliferator-activated receptor γ
PPARγ	Peroxisome proliferator-activated receptor-γ
Prkaa2	5′-AMP-activated protein kinase catalytic subunit α-2
Ptplb	Protein-tyrosine phosphatase-like member B
Ptpn11	Tyrosine-protein phosphatase nonreceptor type 11
RAGE	Receptor for advanced glycation end products
RANKL	Receptor activator of NF-κB ligand
ROS	Reactive oxygen species
SIRT1	Sirtuin
Sirt-1	Silent information regulator sirtuin 1
SLC7A11	Solute carrier family 7 member 11
SMAD	Mothers against decapentaplegic homolog
SOD	Superoxide dismutase
Sorbs1	Sorbin and SH3 domain-containing protein 1
STAT3	Signal transducer and activator of transcription 3
STZ	Streptozotocin
T2DM	Diabetes mellitus type 2
TAC	Total antioxidant capacity
T-AOC	Total antioxidant capacity
TGF-β	Transforming growth factor β (TGF-β)
TIMP-2	Tissue inhibitor of matrix metalloproteinases-2
TLR4	Toll-Like Receptor 4
TNF-α	Tumor necrosis factor
Ttn	Cytoskeletal protein titin
VCAM	Vascular cell adhesion molecule
VEGF	Vascular endothelial growth factor
VEGFR	Vascular endothelial growth factor receptor
XN	Xanthohumol
β-MyHC	Antibodies against myosin heavy chain β
